# Automated magnetic resonance imaging quantification of cerebral parenchymal and ventricular volume following subarachnoid hemorrhage: associations with cognition

**DOI:** 10.1007/s11682-024-00855-0

**Published:** 2024-01-31

**Authors:** Lieke S. Jorna, Sara Khosdelazad, Justyna Kłos, Sandra E. Rakers, Anouk van der Hoorn, Jan Hendrik Potze, Ronald J. H. Borra, Rob J. M. Groen, Jacoba M. Spikman, Anne M. Buunk

**Affiliations:** 1 Department of Neurology, Unit Neuropsychology, University Medical Center Groningen, University of Groningen, Groningen, The Netherlands; 2 Department of Nuclear Medicine, University Medical Center Groningen, Groningen, The Netherlands; 3 Department of Radiology, University Medical Center Groningen, University of Groningen, Groningen, The Netherlands; 4 Department of Neurosurgery, University Medical Center Groningen, University of Groningen, Groningen, The Netherlands; 5 Department of Neurosurgery, Faculty of Medicine Universitas Airlangga, Dr. Soetomo General Academic Hospital, Surabaya, Indonesia

**Keywords:** Subarachnoid hemorrhage, Neuroimaging, Cognition, Magnetic resonance imaging, Neuropsychology

## Abstract

This study aims to investigate cerebral parenchymal and ventricular volume changes after subarachnoid hemorrhage (SAH) and their potential association with cognitive impairment. 17 patients with aneurysmal SAH (aSAH) and 21 patients with angiographically negative SAH (anSAH) without visually apparent parenchymal loss on conventional magnetic resonance imaging (MRI) were included, along with 76 healthy controls. Volumetric analyses were performed using an automated clinical segmentation and quantification tool. Measurements were compared to on-board normative reference database (*n* = 1923) adjusted for age, sex, and intracranial volume. Cognition was assessed with tests for psychomotor speed, attentional control, (working) memory, executive functioning, and social cognition. All measurements took place 5 months after SAH. Lower cerebral parenchymal volumes were most pronounced in the frontal lobe (aSAH: *n* = 6 [35%], anSAH *n* = 7 [33%]), while higher volumes were most substantial in the lateral ventricle (aSAH: *n* = 5 [29%], anSAH *n* = 9 [43%]). No significant differences in regional brain volumes were observed between both SAH groups. Patients with lower frontal lobe volume exhibited significantly lower scores in psychomotor speed (*U* = 81, *p* = 0.02) and attentional control (*t* = 2.86, *p* = 0.004). Additionally, higher lateral ventricle volume was associated with poorer memory (*t* = 3.06, *p* = 0.002). Regional brain volume changes in patients with SAH without visible parenchymal abnormalities on MRI can still be quantified using a fully automatic clinical-grade tool, exposing changes which may contribute to cognitive impairment. Therefore, it is important to provide neuropsychological assessment for both SAH groups, also including those with clinically mild symptoms.

**Supplementary Information:**

The online version contains supplementary material available at 10.1007/s11682-024-00855-0.

## Introduction

A non-traumatic subarachnoid hemorrhage (SAH) is a type of stroke typically caused by the rupture of an aneurysm, known as an aneurysmal subarachnoid hemorrhage (aSAH). In approximately 15% of cases, no structural cause can be identified, termed angiographically negative subarachnoid hemorrhage (anSAH) (Osgood, 2021). SAH can lead to cerebral atrophy, as visible on conventional brain scans, due to the initial bleeding and secondary complications such as rebleeding, hydrocephalus, vasospasm, and delayed cerebral ischemia (Boswell et al., 2013; Gross et al., 2012). Additionally, metabolic alterations post-ictus may lead to permanent changes in capillary morphology, contributing to prolonged tissue hypoxia, inflammation, and atrophy (Østergaard et al., 2013). These detrimental effects on the brain can lead to a decrease in cerebral parenchymal volume and/or increase in ventricular volume.

To date, most brain volume research following SAH has focused on patients with aSAH. A review by Stehouwer et al. (2018). provided strong evidence for total brain volume differences between patients with aSAH and healthy controls (HC). De Bresser et al. (2012) found larger lateral ventricular volumes in patients with aSAH as early as 6 months, while total brain volume was similar to HC. Bendel et al. (2010) demonstrated ventricular and sulcal enlargement together with reduced gray matter volumes one year after aSAH, suggesting of general atrophy. Additionally, long-term ventricular enlargement and smaller parenchymal volume after aSAH are found to be related to delayed cerebral infarction (Bresser et al., 2015). Currently, only one study investigated brain volume loss in eight patients with anSAH, demonstrating significant reductions in intracranial, white matter, whole brain, and hippocampal volume compared to HC (Gama Lobo & Fragata, 2022). In the current study, we aim to investigate cerebral parenchymal and ventricular volume in patients without visually apparent parenchymal loss on conventional MRI, including both aSAH and a larger anSAH cohort.

Cognitive impairment can be considered a sign of brain damage and may be linked to changes in cerebral parenchymal volume and/or ventricular volume. Impairments in memory, attention, executive functioning and social cognition are frequently found both after aSAH and anSAH (Buunk et al., 2016; Burke et al., 2018; Nussbaum et al., 2020). A previous MRI study revealed an association between higher parenchymal lesion volumes and poorer neuropsychological test performance in general intellectual functioning, memory, language, and executive functions twelve months after aSAH (Bendel et al., 2008). Within the same patient group, correlations were found between enlarged cerebral spinal fluid (CSF) spaces and cognitive deficits across multiple domains (Bendel et al., 2010) Hence, it is evident that visible brain damage on MRI after SAH is associated with cognitive impairment. However, it remains to be investigated whether this association is precent in patients with SAH without visual abnormalities on conventional MRI.

*cNeuro cMRI* is a novel automatic clinical MRI quantification tool that performs quantitative volumetric analysis of T1-weighted and FLAIR images (Combinostics Ltd, Tampere, Finland) (Lötjönen et al., 2010). The software, validated in multiple sclerosis (Hänninen et al., 2019; Niiranen et al., 2022) and dementia (Bruun et al., 2019; Lötjönen et al., 2011), provides high quality segmentation in patients without major visual brain damage and provides detailed information about the location of brain volume loss. It therefore seems to be a suitable method for mapping brain volume changes in patients with SAH without visually apparent parenchymal loss.

The aim of this MRI study is twofold. First, to investigate volumetric changes in various brain regions (i.e. cerebral cortex, frontal lobe, temporal lobe, parietal lobe, occipital lobe, medial temporal lobe, cerebral grey matter, lateral ventricles, 3th ventricle, 4th ventricle) in patients with aSAH and anSAH without visually apparent parenchymal loss on conventional MRI. Second, to determine whether lower cerebral parenchymal volume and/or higher ventricular volume are associated with cognitive impairment across multiple domains.

## Methods

### Study design

**Fig. 1:**
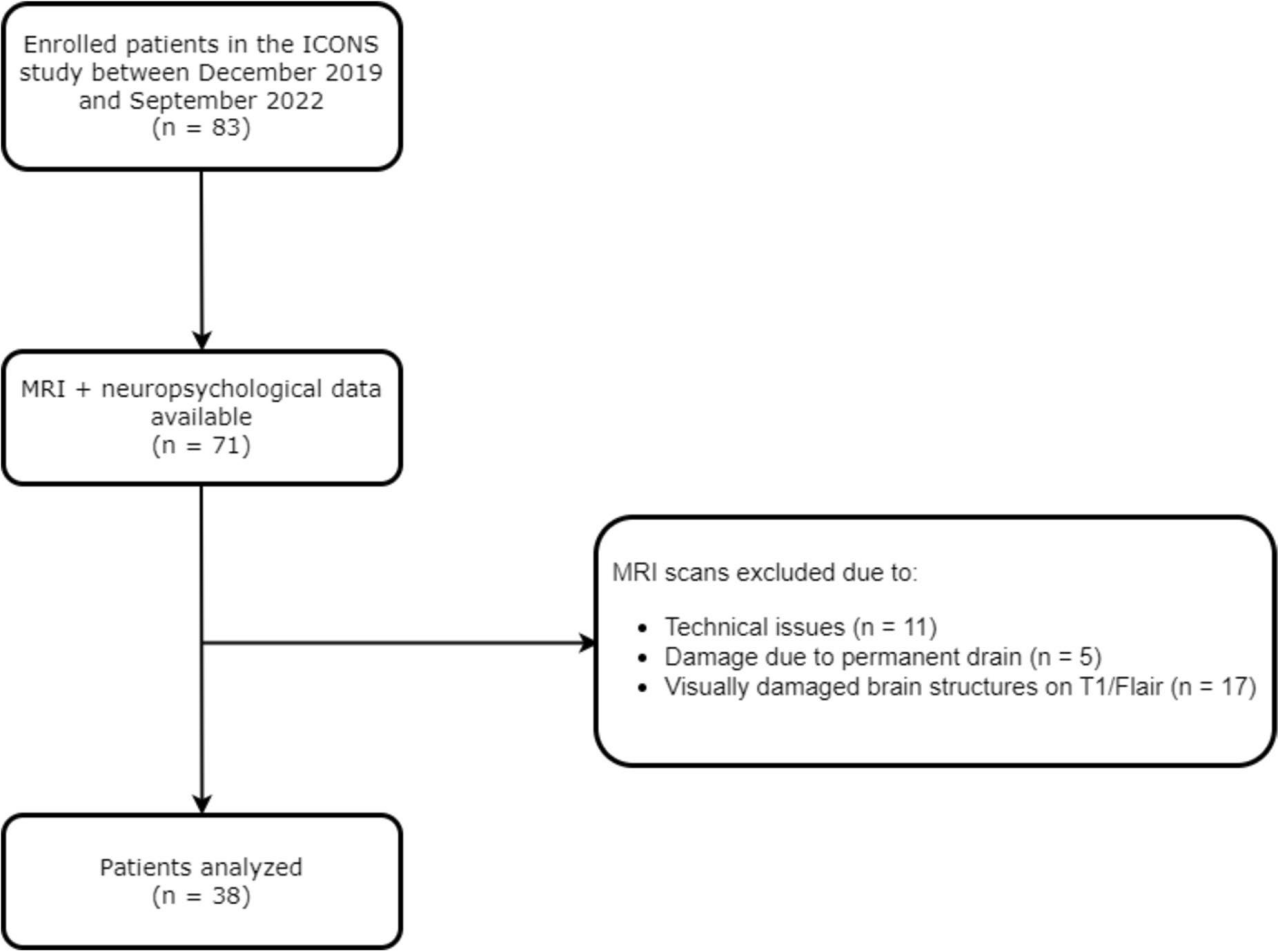
Flowchart illustrating the SAH patients who were included, and whose MRI scans did not exhibit visually apparent incorrect segmentation in cNeuro

This study was part of a larger prospective study, the Imaging, Cognition and Outcome of Neuropsychological functioning after Subarachnoid hemorrhage (ICONS) study, (Khosdelazad et al., 2022) at the University Medical Centre Groningen (UMCG). The study protocol was approved by the Medical Ethical Committee (nr. 2019.346) of the UMCG and was conducted in accordance with the Declaration of Helsinki. For the present study, we included patients diagnosed with aSAH or anSAH between December 2019 and September 2022 without focal areas of cerebral parenchymal loss or gliosis on native T1 and FLAIR images or other factors resulting in visually apparent incorrect segmentation of brain structures (Fig. 1). Exclusion criteria were the presence of serious neurological co-morbidity (e.g. subdural hematoma, previous CVA) or psychiatric disorders. Data regarding demographics (age, sex), clinical characteristics (CSF drainage, SAH type) and clinical condition at admission (World Federation of Neurological Surgeons [WFNS] (Teasdale et al., 1988) were collected from the patients’ medical records. Additionally, 76 HC (HC1) were included for comparative analysis of neuropsychological performance. From this HC1 cohort, a subset (HC2) of 15 participants underwent MRI scans.

### MRI acquisition

Approximately five months post-ictus, MRI imaging was conducted to evaluate potential atrophy on standard clinical scans. This timeframe aligns with the routine follow-up scans in patients with aSAH, to assess the effectiveness of endovascular treatment. MRI data was obtained using a Siemens 3 Tesla scanner (MAGNETOM Prisma, Siemens, Erlangen, Germany) with a standard 64-channel head-neck coil. Patients were scanned according to a standardized scanning protocol as described earlier (Khosdelazad et al., 2022). The sequences that were used for the current study included sagittal 3-dimensional T1-weighted (sagittal slices, voxel size: 0.9 × 0.9 × 0.9 mm, repetition time (TR)/echo time (TE)/time to inversion (TI): 2300/2.31/900 ms) and FLAIR (transversal slices, voxel size: 0.7 × 0.7 × 4.0 mm, TR/TE/TI: 9000/81/2500 ms).

### Brain volumetric analysis

Regional brain volumes were assessed using the FDA and CE approved software *cNeuro cMRI*, developed by Combinostics Ltd in Tampere, Finland (https://www.combinostics.com/cmri/). *cNeuro cMRI* is a validated, medical device intended for clinical use by medical professionals and performs fully automated, objective, visually inspectable segmentation of T1 and FLAIR MRI images, encompassing 133 brain regions. c*Neuro cMRI* automatically compares the extracted volumes to an on-board normative database derived from 1923 healthy individuals (18–94 years, 57% females), with results normalized for age, sex, and head size. The tool provides both numerical (percentile scores) and graphical information, offering insight into whether the volumes for each brain structure in each patient fall within the normal range. Additionally, it provides the percentiles for both left and right-sided structures when applicable, as well as the total volume, which was utilized in this study.

An additional visual quality check of the segmentations was performed by an experienced neurologist with additional imaging training (JK). We focused on the volumes of the following brain regions: frontal lobe, temporal lobe, medial temporal lobe, parietal lobe, occipital lobe, cerebral grey matter, lateral ventricle, inferior lateral ventricle, 3rd ventricle, 4th ventricle, and total cerebral cortex. To enhance clarity, we used the terms ‘lateral ventricle - temporal horn’ instead of *cNeuro’s* ‘inferior lateral ventricle’ and ‘lateral ventricle - frontal horn/body/occipital horn’ instead of ‘lateral ventricle’.

### Neuropsychological assessment

Neuropsychological assessments were performed five months post-ictus, including tests to measure multiple cognitive domains. Psychomotor speed was measured with the Trail Making Test A (TMT-A), (Reitan, 1958) and the Vienna Testing System (VTS) Reaction Time (RT) tasks S1 and S2 (Schuhfried, 2013). Working memory was assessed with the Digit Span Forward and Backward (subtests of the Weschler Adult Intelligence Scale) (Stinissen et al., 1970). Memory was measured with the Dutch version of the Rey Auditory Verbal learning Test (15 Words test, 15WT), (Deelman et al., 1980) immediate recall (IR) and delayed recall (DR). Executive control was assessed with the TMT-B (Reitan, 1958) and the VTS RT S3 and Determination Task (DT) S1 (Schuhfried, 2013). Lastly, social cognition was assessed with The Facial Expressions of Emotion Stimuli and Test (FEEST) (Young et al., 2002).

### Statistical analysis

Statistical analyses were performed using the Statistical Package for the Social Sciences (Version 28.0). Educational level was scored using the Dutch classification system ranging from (1) = no primary school, to (7) = university (Verhage, 1964). Descriptive statistics were used to describe demographic and clinical characteristics. Brain volumes were normalized for age, sex, and head size (percentile values). *cNeuro cMRI* has internal cut-off values to identify atypical brain volume values, compared to the on-board normative database. Cerebral parenchymal volume values below the 10th percentile were categorized as ‘lower’. Ventricular volumes above the 90th percentile were categorized as ‘higher’. Normality assumptions were checked. To test for differences in volume measurements between the total SAH group and HC2, Mann-Whitney U and independent *t*-tests tests were used. Raw scores for neuropsychological measures were compared between the total SAH patient group and HC1, using Mann-Whitney U and independent *t-*tests. Subsequently, raw scores of all neuropsychological measures were transformed into z-scores based on sex, age, and education matched normative data (Lezak et al., 2004). Then, z-scores were computed based on raw scores, averaged within each cognitive domain (excluding social cognition). These z-scores were then compared between patients with and without low cerebral parenchymal volumes or high ventricular volumes in specific brain regions, using Mann-Whitney U and independent t-tests. A significance threshold of *p* < 0.05 (two-tailed) was applied, with Bonferroni-Holm corrections for multiple comparisons.

## Results

Demographic characteristics of included patients and HC can be found in Table 1. No significant differences were found between the two patient groups and HC1, as well as between the two patient groups and HC2, for sex (*X*^*2*^_*HC1*_ = 2.32, *p*_*HC1*_ = 0.31; *X*^*2*^_*HC2*_ = 2.47, *p*_*HC2*_ = 0.48) and age (*F*_*HC1*_ = 0.32, *p*_*HC1*_ = 0.73; *F*_*HC2*_ = 0.34, *p*_*HC2*_ = 0.71).

**Table 1:** Demographic characteristics of patients with aSAH and anSAH and healthy controls

	aSAH (*n* = 17)	anSAH (*n* = 21)	HC1 (*n* = 76)	HC2 (*n* = 15)
Sex, women, *n*(%)	9(53%)	10(48%)	49(36%)	9(60%)
Age at time of SAH, years (M ± SD)	55.5 ± 15.5	53.1 ± 9.9	54.4 ± 7.7	55.8 ± 5.8
Educational level (M ± SD)	5 ± 1.0	5 ± 0.8	5.8 ± 0.8	5.6 ± 0.5
Time at MRI since SAH in months (M ± SD)	5 ± 0.9	5 ± 0.9		
WFNS				
Low (1–3)	16(94%)	21(100%)		
High (4–5)	1(6%)	0		
External CSF drainage (ventricular/lumbar)	3(18%)	3(14%)		

*SAH * subarachnoid hemorrhage, *aSAH *aneurysmal subarachnoid hemorrhage, *anSAH *angiographically negative subarachnoid hemorrhage, *HC *healthy controls, *SD *standard deviation, *WFNS *World Federation of Neurological Surgeons, *CSF *cerebrospinal fluid

### Volumetric analysis

In Table 2, percentages of patients and HC with lower or, in case of the ventricles, higher volumetric measurements as compared to the on-board normative database are depicted for multiple brain regions. In both patients with aSAH and anSAH, lower regional cerebral parenchymal volume was most profound in the frontal lobe. Higher ventricular volume was most frequent in the lateral ventricle - temporal horn and fourth ventricle after aSAH and in the lateral ventricle - frontal horn/body/occipital horn after anSAH.

**Table 2:** Numbers and percentages of patients with SAH with significantly lower (percentile < 10) regional cerebral parenchymal volume and higher (percentile > 90) ventricular volume compared to an on-board normative database

	aSAH (*n* = 17)	anSAH (*n* = 21)	HC2 (*n* = 15)
Cerebral parenchymal volume			
Frontal lobe	6(35%)	7(33%)	3(20%)
Temporal lobe	1(6%)	3(14%)	0
Parietal lobe	2(12%)	1(5%)	1(7%)
Occipital lobe	1(6%)	4(19%)	3(20%)
Medial Temporal Lobe	4(24%)	2(10%)	1(7%)
Cerebral GM	3(18%)	4(19%)	2(13%)
Total cerebral cortex	2(12%)	4(19%)	2(13%)
Ventricular volume			
Lateral ventricle - frontal horn/body/occipital horn	5(29%)	9(43%)	1(7%)
Lateral ventricle - temporal horn	7(41%)	3(24%)	0
3rd ventricle	5(29%)	6(29%)	1(7%)
4th ventricle	7(41%)	5(24%)	1(7%)

*aSAH *aneurysmal subarachnoid hemorrhage, *anSAH *angiographically negative subarachnoid hemorrhage, *GM *grey matter, *HC *healthy controls

We found no significant differences in cerebral parenchymal or ventricular volume between patients with aSAH and anSAH (all *ps* > 0.05). For this reason, data for both SAH groups were pooled for further analysis. When comparing all regions between the total SAH group and HC2 group, we found significant differences in the lateral ventricle - frontal horn/body/occipital horn, lateral ventricle - temporal horn, and 3rd ventricle (Supplementary Table 1). Patients with SAH had higher volumes in all these regions. Although not significant, patients with SAH had lower volumes in all cerebral parenchymal regions, except for cerebral gray matter and the occipital lobe.

### Neuropsychological analysis

Table 3 presents mean scores on all neuropsychological tests, tested for differences between patients with SAH and HC1. We found significant differences on all measures, except for the TMT-A, Digit Span Backward, and VTS RT S3.

**Table 3:** Differences between patients with SAH (both aSAH and anSAH) and healthy controls on neuropsychological tests

	SAH patients (*n* = 38)	HC1 (*n* = 76)		
	M ± SD	M ± SD	*t/Z*^*a*^	*p*
Psychomotor speed				
TMT-A	36 ± 15.4	30.8 ± 9.8	1125.5	0.06
VTS RT S1	329.9 ± 86.5	277.8 ± 49.7	845.5	< 0.001*
VTS RT S2	271.1 ± 85	233.8 ± 45.8	989.5	0.01*
Working memory				
Digit Span Forward	8.2 ± 1.9	9.2 ± 2.1	1033.5	0.01*
Digit Span Backward	7.8 ± 1.7	8.5 ± 1.7	1166.5	0.09
Memory				
15 Words Test - IR	41.3 ± 8.8	46.1 ± 9.9	1028.5	0.01*
15 Words Test - DR	8.0 ± 2.8	9.7 ± 2.5	940.5	0.002*
Executive control				
TMT-B	87.1 ± 53	61 ± 19.9	945	0.003*
VTS RT S3	491.8 ± 100.8	454.1 ± 76.9	1154	0.1
VTS DT S1	195.6 ± 38.1	227.4 ± 27.2	716.5	< 0.001*
Social cognition				
FEEST	45.8 ± 5.6	49.1 ± 4.9	-3.2	0.002*

*SAH *subarachnoid hemorrhage, *HC *healthy controls, *TMT-A *Trail Making Test part A, *VTS RT S1 *Vienna Testing System Reaction Time S1, *VTS RT S2 *Vienna Testing System Reaction Time S2, *IR *immediate recall, *DR *delayed recall, *TMT-B *Trail Making Test part B, *VTS RT S3 *Vienna Testing System Reaction Time S3, *VTS DT S1* Vienna Testing System Determination Task S1, *FEEST *Facial Expression of Emotional Stimuli Test, *M *mean, *SD *standard deviation, *t *test statistic, *p *p-value

^a^Independent t-test for FEEST

*significant at < 0.05

Associations between cognition and two specific brain regions where significant volumetric changes were most commonly observed (i.e. frontal lobe and lateral ventricle – frontal horn/body/occipital horn), were investigated. Table 4 indicates that patients with SAH with lower frontal lobe volume demonstrate significantly lower scores for psychomotor speed and executive control compared to patients with normal frontal lobe volume, suggesting slower cognitive processing and worse executive control.

**Table 4:** Differences between patients with SAH (both aSAH and anSAH) with and without lower frontal lobe parenchymal volume on neuropsychological measures

	Lower frontal lobe volume (*n*=13)	Normal frontal lobe volume (*n*=25)		
	M ± SD	M ± SD	*t*/*U*^a^	*p*
Psychomotor speed	-0.95 ± 0.71	-0.29 ± 1.01	81.0	0.02*
Working memory	-0.51 ± 0.85	-0.22 ± 0.75	1.10	0.14
Memory	-0.46 ± 0.69	-0.41 ± 0.72	0.26	0.40
Executive control	-0.36 ± 0.46	0.08 ± 0.42	2.86	0.004*
Social cognition	-.30 ± 0.97	-0.55 ± 0.73	137.5	0.56

The neuropsychological measures are composite scores

*M *mean, *SD *standard deviation, *t *test statistic for independent t-test, *p *p value

^a^Mann-Whitney U test for psychomotor speed, social cognition

*significant at < 0.05

Table 5 presents the differences in neuropsychological measures across multiple domains between patients with SAH with higher lateral ventricle – frontal horn/body/occipital horn volume and those with normal volumes. Patients with higher volume in this region demonstrated lower memory scores, indicating memory impairment. Among those with higher lateral ventricle - frontal horn/body/occipital horn volumes, 14% (*n* = 2) had a temporary drain during acute hospital admission. No significant differences were found for the lateral ventricle - temporal horn, 3rd, and 4th ventricle regarding neuropsychological measures between patients with and without higher volumes.

**Table 5:** Differences between patients with SAH (both aSAH and anSAH) with and without higher lateral ventricle – frontal horn/body/occipital horn volume on neuropsychological measures

	higher volume (*n*=14)	Normal volume (*n*=24)		
	M ± SD	M ± SD	*t*/*U*^a^	*p*
Psychomotor speed	-0.50 ± 1.12	0.17 ± 0.89	159.5	0.59
Working memory	-0.18 ± 0.93	-0.40 ± 0.70	-0.85	0.20
Memory	-0.84 ± 0.50	-0.19 ± 0.70	3.06	0.002*
Executive control	-0.03 ± 0.64	-0.09 ± 0.37	-0.33	0.37
Social cognition	-0.46 ± 0.85	-0.47 ± 1.06	143.5	0.96

The neuropsychological measures are composite scores

*M *mean, *SD *standard deviation, *t *test statistic for independent t-test, *p *p value

^a^Mann-Whitney U test for psychomotor speed, social cognition

*significant at < 0.05

## Discussion

This study suggests that quantitatively measured differences in cerebral parenchymal and ventricular volume can be detected in patients with aSAH and anSAH, even when no visible parenchymal loss is observed on conventional MRI. Cognitive impairments were evident across all domains (psychomotor speed, memory, executive control, and social cognition). In both SAH groups, lower regional cerebral parenchymal volume was most frequently observed in the frontal lobe, associated with poorer performance in psychomotor speed and executive control compared to patients with normal frontal lobe volume. Moreover, higher lateral ventricle volume was associated with worse memory performance in both SAH groups.

Patients with lower cerebral parenchymal volume, compared to the on-board normative database of the automatic MRI quantification tool, were found across all measured regions, with the frontal lobe being most affected. 35% of patients with aSAH and 33% of patients with anSAH demonstrated lower frontal lobe volume. Our findings align with prior aSAH studies (Bendel et al., 2008) and contribute to the literature by demonstrating lower frontal lobe volume in patients with anSAH as well. No significant differences in regional cerebral parenchymal volumes were found between the two SAH groups in any of the measured regions, suggesting that endovascular treatment received by patients with aSAH does not influence the extent of brain damage. These results provide additional support for the role of metabolic changes in neurodegeneration after SAH (Østergaard et al., 2013). In line with previous research in patients with anSAH (Gama Lobo & Fragata, 2022), this study challenges the assumption of a favorable outcome in this patient group.

When comparing the total SAH group to HC, significantly higher volumes in the lateral ventricle - frontal horn/body/occipital horn, lateral ventricle - temporal horn, and 3rd ventricle were identified in patients as early as 5 months post-ictus. Although cerebral parenchymal volumes were generally lower in patients, excluding gray matter volume and occipital lobe volume, these differences lacked statistical significance.

Previous research demonstrated compromised psychomotor speed and executive control after both aSAH (Buunk et al., 2016; Nussbaum et al., 2020) and anSAH (Burke et al., 2018; Khosdelazad et al., 2023). Kochunov et al. (2010) showed that psychomotor speed is correlated with frontal lobe functioning in healthy older adults. Additionally, a recent review provides abundant proof of the relation between executive control and prefrontal cortex functioning (Friedman & Robbins, 2022). Our study is the first to find an association between lower psychomotor speed, worse executive control, and lower frontal lobe volume, indicating that lower cerebral parenchymal volume in this area negatively impacts cognitive functioning. In addition, we investigated social cognition in relation to frontal lobe volume in patients with SAH, which had not been previously explored. Although prefrontal cortex regions play an important role in social cognition, (Friedman & Robbins, 2022; Forbes & Grafman, 2010) this study did not find a relation between lower frontal lobe volume and poorer performance in this cognitive domain. Different aspects of social cognition are related to specific regions within the prefrontal cortex; the orbitofrontal cortex, medial and ventromedial prefrontal cortex seem particularly important in emotion recognition (Forbes & Grafman, 2010; Sabatinelli et al., 2011). It is possible that the impact of lower frontal lobe volume was minimal in these specific brain regions, thereby limiting its effects on emotion recognition.

Furthermore, higher ventricular volumes were found in a substantial proportion of patients in both SAH groups. Ventricular enlargement can either be a consequence or a cause of brain atrophy (Li et al., 2018). When brain tissue volume decreases due to atrophy, a compensatory response often involves the expansion of the ventricles. On the other hand, SAH-related elevated intracranial pressure can result in enlargement of ventricular system/pericerebral CSF-spaces. Excess CSF can compress and distort surrounding brain structures, impeding blood supply and causing tissue damage and atrophy. Within this study, 18% of patients with aSAH and 14% of patients with anSAH received treatment for acute hydrocephalus through (temporary) external CSF drainage in the acute stage following SAH. None of the patients developed a permanent CSF resorption disorder, indicating effective temporary drainage. Previous research found higher CSF/ICV ratios in patients with SAH with at least one neuropsychological deficit compared to those without any deficits, indicating a potential relationship between increased ventricular volume and neuropsychological impairments (Bendel et al., 2010). Our study indicates that patients with higher lateral ventricle - frontal horn/body/occipital volumes exhibited worse performance on memory tests, while performance in other cognitive domains did not show significant differences. This association is not surprising, given that the hippocampus, which plays a major role in learning and memory, lines the lateral ventricle. Enlargement of the lateral ventricles can affect neighboring structures, including the hippocampus, which can result in memory difficulties. Previous research has also found associations between increased lateral ventricular volume and worse memory performance in older adults (Harrison et al., 2012) and in subjects with mild cognitive impairment (Rogne et al., 2016). The current study indicates that this association may also be observed in patients with aSAH and anSAH.

While this study provides valuable insights, certain limitations should be acknowledged. First, the absence of imaging during the acute phase post-ictus precluded longitudinal assessments of atrophy rates over time. Future research should incorporate such assessments to enhance understanding of dynamic changes in brain structure following aSAH and anSAH. Second, no distinction was made within the anSAH patient group regarding a perimesencephalic or a diffuse blood distribution. Investigating this distinction in future research could provide valuable insights, considering that secondary complications are more common in patients with a diffuse bleed pattern (Gross et al., 2012). Lastly, we excluded patients with aSAH whose aneurysms were clipped due to the expected presence of artifacts in the anatomical MRI series and as a result in the segmentation. It would be interesting to investigate the impact of surgical intervention on brain volume in the future.

## Conclusions

In conclusion, this study suggests that decreases in regional cerebral parenchymal volume and/or enlargements of ventricular volume can occur after aSAH and anSAH, even without evident parenchymal loss on conventional brain scans. More importantly, lower frontal brain volume and higher lateral ventricular volume were associated with reduced cognitive functioning, which may negatively affect daily life functioning. Therefore, our study highlights the significance of conducting neuropsychological assessment for both patients with aSAH and anSAH, including those patients with clinically mild symptoms. Such assessments can provide valuable insights into cognitive impairments and guide appropriate care and interventions.

### Supplementary information

Below is the link to the electronic supplementary material.

ESM 1 (DOCX 16.5 KB)

## Data Availability

Depending on the type of data and associated privacy regulations, data will be shared upon reasonable request.

## References

[CR1] Bendel P, Koivisto T, Äikiä M, Niskanen E, Könönen M, Hänninen T (2010). Atrophic enlargement of CSF volume after subarachnoid hemorrhage: Correlation with neuropsychological outcome. American Journal of Neuroradiology.

[CR2] Bendel P, Koivisto T, Könönen M, Hänninen T, Hurskainen H, Saari T (2008). MR imaging of the brain 1 year after aneurysmal subarachnoid hemorrhage: Randomized study comparing surgical with endovascular treatment. Radiology.

[CR3] Boswell S, Thorell W, Gogela S, Lyden E, Surdell D (2013). Angiogram-negative subarachnoid hemorrhage: Outcomes Data and Review of the literature. Journal of Stroke and Cerebrovascular Diseases : The Official Journal of National Stroke Association.

[CR4] Bruun M, Koikkalainen J, Rhodius-Meester HFM, Baroni M, Gjerum L, van Gils M (2019). Detecting frontotemporal dementia syndromes using MRI biomarkers. NeuroImage Clinical [Internet].

[CR5] Burke T, Hughes S, Carr A, Javadpour M, Pender N (2018). A systematic review of cognitive outcomes in Angiographically negative subarachnoid haemorrhage. Neuropsychology Review.

[CR6] Buunk AM, Groen RJM, Veenstra WS, Metzemaekers JDM, van der Hoeven JH, van Dijk JMC (2016). Cognitive deficits after aneurysmal and angiographically negative subarachnoid hemorrhage: Memory, attention, executive functioning, and emotion recognition. Neuropsychology.

[CR7] de Bresser J, Schaafsma JD, Luitse MJA, Viergever MA, Rinkel GJE, Biessels GJ (2015). Quantification of structural cerebral abnormalities on MRI 18 months after aneurysmal subarachnoid hemorrhage in patients who received endovascular treatment. Neuroradiology.

[CR8] de Bresser J, Vincken KL, Kaspers AJ, Rinkel GJ, Viergever MA, Biessels GJ (2012). Quantification of cerebral volumes on MRI 6 months after aneurysmal subarachnoid hemorrhage. Stroke.

[CR9] Deelman, B. G., Brouwer, W. H., Van Zomeren, A. H., & Saan, R. J. (1980). Functiestoornissen na trauma capitis. In A .Jennekens-Schinkel, J. Diamant, H. Diesfeldt, R. Haaxma (EdS.). *Neuropsychologie in Nederland. Deventer, the Netherlands: Van Loghum Slaterus*, pp. 253–281. 35.

[CR10] Forbes CE, Grafman J (2010). The role of the human prefrontal cortex in social cognition and moral judgment *. Annual Review of Neuroscience.

[CR11] Friedman NP, Robbins TW (2022). The role of prefrontal cortex in cognitive control and executive function. Neuropsychopharmacology : Official Publication of the American College of Neuropsychopharmacology.

[CR12] Gama Lobo G, Fragata I (2022). Long-term global and focal cerebral atrophy in perimesencephalic subarachnoid hemorrhage - a case–control study. Neuroradiology.

[CR13] Gross BA, Lin N, Frerichs KU, Du R (2012). Vasospasm after spontaneous angiographically negative subarachnoid hemorrhage. Acta Neurochirurgica. Supplementum.

[CR14] Hänninen K, Viitala M, Paavilainen T, Karhu JO, Rinne J, Koikkalainen J (2019). Thalamic atrophy without whole brain atrophy is associated with absence of 2-year NEDA in multiple sclerosis. Frontiers in Neurology.

[CR15] Harrison TM, Weintraub S, Mesulam MM, Rogalski E (2012). Superior memory and higher cortical volumes in unusually successful cognitive aging. Journal of the International Neuropsychological Society.

[CR16] Khosdelazad S, Jorna LS, Groen RJM, Rakers SE, Timmerman ME, Borra RJH (2022). Investigating Recovery after Subarachnoid Hemorrhage with the imaging, Cognition and Outcome of Neuropsychological Functioning after Subarachnoid Hemorrhage (ICONS) study: Protocol for a longitudinal, prospective cohort study. JMIR Research Protocols.

[CR17] Khosdelazad S, Jorna LS, Rakers SE, Kof R, Groen RJM, Spikman JM (2023). Long-term course of cognitive Functioning after Aneurysmal and Angiographically negative. Neurosurgery.

[CR18] Kochunov P, Coyle T, Lancaster J, Robin D, Hardies J, Kochunov V (2010). Processing speed is correlated with cerebral health markers in the frontal lobes as quantified by neuro-imaging. NeuroImage.

[CR19] Lezak MD, Howieson DB, Loring DW, Hannay HJ, Fischer JS (2004). Neuropsychological Assessment.

[CR20] Li X, Ba M, Pin K, Mathotaarachchi S (2018). Characterizing biomarker features of cognitively normal individuals with ventriculomegaly. Alzheimers Dement (Amst).

[CR21] Lötjönen J, Wolz R, Koikkalainen J, Julkunen V, Thurfjell L, Lundqvist R (2011). Fast and robust extraction of hippocampus from MR images for diagnostics of Alzheimer’s disease. NeuroImage.

[CR22] Lötjönen JM, Wolz R, Koikkalainen JR, Thurfjell L, Waldemar G, Soininen H (2010). Fast and robust multi-atlas segmentation of brain magnetic resonance images. NeuroImage.

[CR23] Niiranen M, Koikkalainen J, Lötjönen J, Selander T, Cajanus A, Hartikainen P (2022). Grey matter atrophy in patients with benign multiple sclerosis. Brain and Behavior: A Cognitive Neuroscience Perspective.

[CR24] Nussbaum ES, Mikoff N, Paranjape GS (2020). Cognitive deficits among patients surviving aneurysmal subarachnoid hemorrhage. A contemporary systematic review. British Journal of Neurosurgery.

[CR25] Osgood ML (2021). Aneurysmal Subarachnoid Hemorrhage: Review of the pathophysiology and management strategies. Current Neurology and Neuroscience Reports.

[CR26] Østergaard L, Aamand R, Karabegovic S, Tietze A, Blicher JU, Mikkelsen IK (2013). The role of the microcirculation in delayed cerebral ischemia and chronic degenerative changes after subarachnoid hemorrhage. Journal of Cerebral Blood Flow and Metabolism.

[CR27] Reitan RM (1958). Validity of the trail making test as an Indicator of Organic Brain damage. Perceptual and Motor Skills.

[CR28] Rogne S, Vangberg T, Eldevik P, Wikran G, Mathiesen EB, Schirmer H (2016). Magnetic resonance volumetry: Prediction of subjective memory complaints and mild cognitive impairment, and associations with Genetic and Cardiovascular Risk factors. Dementia and Geriatric Cognitive Disorders.

[CR29] Sabatinelli D, Fortune EE, Li Q, Siddiqui A, Krafft C, Oliver WT (2011). Emotional perception: Meta-analyses of face and natural scene processing. NeuroImage.

[CR30] Schuhfried G (2013). Vienna test system: Psychological assessment.

[CR31] Stehouwer, B. L., van der Kleij, L. A., Hendrikse, J., Rinkel, G. J. E., & De Vis, J. B. (2018). *Magnetic resonance imaging and brain injury in the chronic phase after aneurysmal subarachnoid hemorrhage: A systematic review,* vol. 13, International Journal of Stroke. SAGE Publications Inc., pp. 24–34. 10.1177/1747493017730781 28920537

[CR32] Stinissen, J., Willems, P. J., Coetsier, P., & Hulsman, W. L. L. (1970). *Handleiding Bij De Nederlandstalige bewerking Van De Wechsler Adult Intelligence Scale (WAIS) [manual of the Dutch edition of the WAIS]*. Swets & Zeitlinger.

[CR33] Teasdale GM, Drake CG, Hunt W, Kassell N, Sano K, Perat B (1988). A universal subarachnoid haemorrhage scale: Report of a committee of the world federation of neurosurgical societies.

[CR34] Verhage, F. (1964). *Intelligence and age: Research in Dutch persons between 12 and 77 years old [Dutch]*. Van Gorcum Assen.

[CR35] Young A, Perrett D, Calder A, Sprengelmeyer R, Ekman P (2002). Facial expressions of emotion: Stimuli and tests (FEEST).

