# Supplementary material for: Automated magnetic resonance imaging quantification of cerebral parenchymal and ventricular volume following subarachnoid hemorrhage: associations with cognition
Source: Brain Imaging Behav. 2024 Jan 31;18(2):421–9. doi: 10.1007/s11682-024-00855-0 (PMC10830824; doi:10.1007/s11682-024-00855-0)
Supplement: Supplementary file 1 — (DOCX 16.5 KB) [file 11682_2024_855_MOESM1_ESM.docx]

**Supplementary Table 1.** Differences between the total SAH group and HC2 group in cerebral parenchymal and ventricular volumes

|  | SAH (*n*=38) | HC2 (*n*=15) | *t/U^b^* | *p* |
| --- | --- | --- | --- | --- |
| **Cerebral parenchymal volume** | **M±SD^a^** | **M±SD^a^** |  |  |
| Frontal lobe | 29.4±29.5 | 35.0±26.8 | 240.0 | 0.37 |
| Temporal lobe | 46.9±28.7 | 49.7±26.3 | -0.33 | 0.75 |
| Parietal lobe | 54.4±29.4 | 62.2±26.7 | 241.0 | 0.39 |
| Occipital lobe | 56.0±34.7 | 40.5±35.6 | 222.0 | 0.21 |
| Medial Temporal Lobe | 45.5±30.0 | 51.3±22.0 | -0.68 | 0.50 |
| Cerebral GM | 40.6±30.8 | 38.4±27.7 | 276.0 | 0.86 |
| Total cerebral cortex | 40.3±30.0 | 41.7±28.5 | 275.0 | 0.84 |
| **Ventricular volume** | **M±SD^a^** | **M±SD^a^** |  |  |
| Lateral ventricle - frontal horn/body/occipital horn | 67.5±29.5 | 47.5±20.3 | 175.0 | **0.03*** |
| Lateral ventricle - temporal horn | 62.5±28.1 | 34.1±21.8 | 120.0 | **0.001*** |
| 3^th^ ventricle | 59.8±32.4 | 36.2±34.3 | 166.0 | **0.02*** |
| 4^th^ ventricle | 63.8±32.3 | 54.5±36.0 | 216.0 | 0.17 |

*Note.* SAH = subarachnoid hemorrhage; HC = healthy controls; GM = grey matter; t/U = test statistic

^a^ Means and standard deviations are percentile scores

^b^ Independent t-test for: temporal lobe and medial temporal lobe

* Significant after Bonferroni-Holm corrections
